# Effects of Gold Nanorods on Imprinted Genes Expression in TM-4 Sertoli Cells

**DOI:** 10.3390/ijerph13030271

**Published:** 2016-03-01

**Authors:** Beilei Yuan, Hao Gu, Bo Xu, Qiuqin Tang, Wei Wu, Xiaoli Ji, Yankai Xia, Lingqing Hu, Daozhen Chen, Xinru Wang

**Affiliations:** 1 State Key Laboratory of Reproductive Medicine, Institute of Toxicology, Nanjing Medical University, Nanjing 211166, China; yuanbeilei@163.com (B.Y.); kobegu08@163.com (H.G.); bxu126@126.com (B.X.); xlji126@126.com (X.J.); yankaixia@njmu.edu.cn (Y.X.); 2 Key Laboratory of Modern Toxicology of Ministry of Education, School of Public Health, Nanjing Medical University, Nanjing 211166, China; 3 State Key Laboratory of Reproductive Medicine, Department of Obstetrics, Nanjing Maternity and Child Health Care Hospital Affiliated to Nanjing Medical University, Nanjing 210004, China; t19871004@sina.com; 4 State Key Laboratory of Reproductive Medicine, Wuxi Maternal and Child Health Care Hospital Affiliated to Nanjing Medical University, Wuxi 214002, China; lqhu126@126.com (L.H.); chendaozhen@163.com (D.C.)

**Keywords:** GNRs, TM-4 Sertoli cells, imprinted genes, reproductive toxicity

## Abstract

Gold nanorods (GNRs) are among the most commonly used nanomaterials. However, thus far, little is known about their harmful effects on male reproduction. Studies from our laboratory have demonstrated that GNRs could decrease glycine synthesis, membrane permeability, mitochondrial membrane potential and disrupt blood-testis barrier factors in TM-4 Sertoli cells. Imprinted genes play important roles in male reproduction and have been identified as susceptible loci to environmental insults by chemicals because they are functionally haploid. In this original study, we investigated the extent to which imprinted genes become deregulated in TM-4 Sertoli cells when treated with low dose of GNRs. The expression levels of 44 imprinted genes were analyzed by quantitative real-time PCR in TM-4 Sertoli cells after a low dose of (10 nM) GNRs treatment for 24 h. We found significantly diminished expression of *Kcnq1*, *Ntm*, *Peg10*, *Slc22a2*, *Pwcr1*, *Gtl2*, *Nap1l5*, *Peg3* and *Slc22a2*, while *Plagl1* was significantly overexpressed. Additionally, four (*Kcnq1*, *Slc22a18*, *Pwcr1* and *Peg3*) of 10 abnormally expressed imprinted genes were found to be located on chromosome 7. However, no significant difference of imprinted miRNA genes was observed between the GNRs treated group and controls. Our study suggested that aberrant expression of imprinted genes might be an underlying mechanism for the GNRs-induced reproductive toxicity in TM-4 Sertoli cells.

## 1. Introduction

In recent decades, gold nanorods (GNRs) have been investigated extensively for clinical imaging [1], treatment [2], drug delivery [3] and cancer therapy [4,5]. However, whether GNRs have reproductive toxicity has aroused wide concern. Apart from cytotoxicity [6]，hepatotoxicity [7] and kidney oxidative damage in mice [8] and cells [9,10], spermatotoxicity of GNRs has been also reported [11]. In our recent study, we investigated the impacts of GNRs on the metabolomics changes in TM-4 Sertoli cells. We found that GNRs treatment could affect male reproduction via a decrease in glycine synthesis, membrane permeability, mitochondrial membrane potential and disrupt blood-testis barrier factors in TM-4 Sertoli cells [12].

The term imprinted gene refers to the monoallelic expression of genes according to the parent of origin [13,14]. This imprinting phenomena is a good example of how multiple epigenetic processes act together via DNA methylation, histone modification and chromatin-modeling elements like non-coding RNAs [13,14]. Genomic imprinting is acquired during gametogenesis when genome-wide epigenetic remodeling occurs and is maintained during development [15]. Imprinting is erased in the primordial germ cells and new methylation imprints are established according to the sex of germ line [16,17]. Imprinted genes are maternally marked in the mature oocyte and paternally marked in the sperm [18]. Many imprinted genes were found in clusters and possess imprinting control regions (ICR) [19,20]. Recent studies have demonstrated that imprinted genes were tightly related to spermatogenesis [21], development of placenta [19], fetal growth [13] and cancer [22]. Previous studies have found that aberrant DNA methylation in spermatozoa was significantly associated with male infertile [21,23]. Besides, other studies have showed that chemical exposure could disrupt the expression and function of imprinted genes [20,24]. However, whether GNRs could affect the expression of imprinted genes—which is important for male reproduction—remains largely undetermined. The aim of this study was to investigate whether GNRs treatment could alter the expression of imprinted genes in TM-4 Sertoli cells.

According to two public databases for imprinted genes (www.geneimprint.com and http://igc.otago.ac.nz/home.html) [19], one hundred and twenty-two and 95 imprinted genes have been identified in mouse and human respectively. Homologous genes were considered as playing similar roles in functional regulation. Thus, in the present study, 44 homologous imprinted genes between mouse and human were finally selected for the expression level analysis in TM-4 Sertoli cells treated with a low dose of GNRs.

## 2. Materials and Methods 

### 2.1. Chemicals and Regents

**Figure 1:**
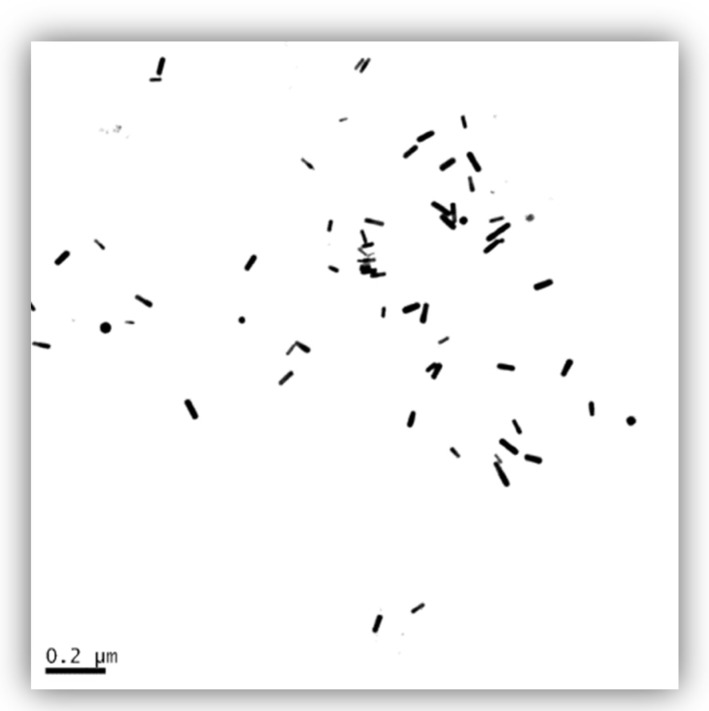
Transmission electron microscopy image of gold nanorods (GNRs).

GNRs with dimensions of (9–11) nm × (34–42) nm were obtained from Sigma-Aldrich (Sigma Chemical Co., Saint Louis, MO, USA). Zeta potential (mV) is 23.4 ± 2.39. Dimethyl sulfoxide (DMSO), bovine serum albumin (BSA) and diethylpyrocarbonate (DEPC) were also purchased from Sigma-Aldrich (Sigma Chemical Co., St. Louis, MO, USA). DMEN medium, fetal bovine serum (FBS), streptomycin sulfate, penicillin G sodium and phosphate-buffered saline with Ca^2+^ and Mg^2+^ (PBS) were obtained from Gibco BRL (Grand Island, NY, USA). All chemicals were of analytical grade. GNRs were stored at 4 °C, and then diluted to 10 nM in culture medium immediately before use. GNRs were well dispersed in the medium and no aggregation was found (Figure 1). 

### 2.2. Cell Culture and GNRs Treatment

TM-4 Sertoli cells were purchased from ATCC (Manassas, VA, USA). The cell line was cultured in growth medium DMEM supplemented with 10% fetal bovine serum (FBS), 100 μg/mL streptomycin and 100 U/mL penicillin in an atmosphere of 5% CO_2_ at 37 °C. In our previous study, we found that GNRs treatment did not affect cell viability at 10 nM, while GNRs with a higher dose caused decreased cell viability [12]. Neither exposure to GNR-10 nM for 24 h nor 48 h induced cytotoxic effects. In the present study, the final concentrations of GNRs treated for TM-4 Sertoli cells were 0 and 10 nM. The freshly diluted GNRs of the two concentrations were administered when the cell confluency reached up to 50%, and the cells were treated for 24 h.

### 2.3. Characteristics of GNRs

The images of transmission electron microscopy (TEM) were recorded using a Jeol JEM-2011 TEM equipped with a Gatan Dual Vision600 CCD.

### 2.4. Total RNA Isolation

Total RNAs were isolated from TM-4 Sertoli cells using the TRIzol reagent (Invitrogen Life Technologies Co, Carlsbad, CA, USA) following the manufacturer’s instructions. The concentration and purity of RNA were determined spectrophotometrically by measuring its optical density (Abs 260/280 > 2.0; Abs 260/230 > 1.8) using Nano Drop ND-1000 Spectrophotometer (Thermo Scientific, Wilmington, DE, USA). Samples were stored at –80 °C until analysis.

### 2.5. Real-Time PCR

cDNA synthesis was performed with 1 μg total RNA using PrimeScript™ RT Master Mix (Perfect Real Time) and PrimeScript™ RT reagent Kit (Perfect Real Time) according to the manufacturer’s instructions (TaKaRa, Dalian, China). All the primers were synthesized by Invitrogen (Shanghai, China). The reverse primers of imprinted genes were listed in Table S1.

### 2.6. Expression Level of Imprinted Genes

The relative expression levels of all tested imprinted genes were determined by quantitative real-time PCR assay (qRT-PCR) using SYBR^®^ Premix Ex Taq™ (Tli RNaseH Plus) according to the manufacturer’s instructions (TaKaRa, Dalian, China). Real-time PCR was performed using the ABI Prism 7900HT (Applied Biosystems, Foster City, CA, USA). The primers used were listed in Table S1. Real-time PCR was performed in duplicate for each sample, and the specificity of PCR product was estimated using the dissociation curve. *Gapdh* and U6 were used as references for mRNA and miRNA relative expression level respectively, and the values were showed by △Ct, all the experiments were repeated for four times.

### 2.7. Statistical Analysis

Dates were presented as the mean ± SEM unless otherwise stated. All dates were analyzed using PASW statistics version 18.0 (SPSS Inc., IBM Corporation, Chicago, IL, USA). We selected *t*-test to compare the different △Ct between the control group and the treated group. Fisher’s Exact Test was chosen to test the difference between rates. The correlation between the differentially expressed genes was investigated using the Pearson correlation test. *p* < 0.05 was regarded as statistical significance. Figures were made using the GraphPad Prism 5 software.

## 3. Results

### 3.1 Expression of Imprinted Genes in Normal and GNRs Treated Group

**Figure 2:**
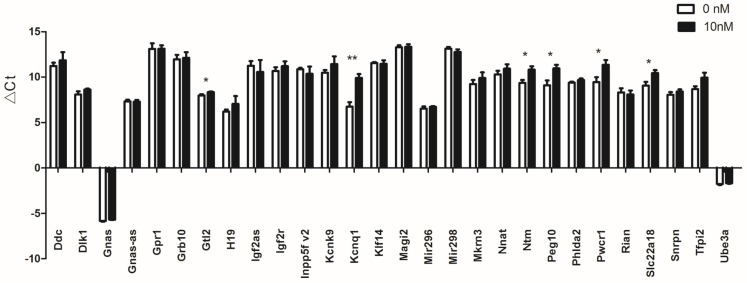
Expression profile of 28 imprinted genes both expressed in control and GNRs treated TM-4 cells. Data are expressed as the mean ± SEM. X axis indicates the names of the imprinted genes both expressed in control and GNRs treated TM-4 cells. Y axis indicates the delta CT values for each imprinted genes (△Ct = Ct _imprinted gene_– Ct _*gapdh*/U6_). Sample number = 4, * *p* < 0.05; ** *p* < 0.01 *vs.* control.

**Figure 3:**
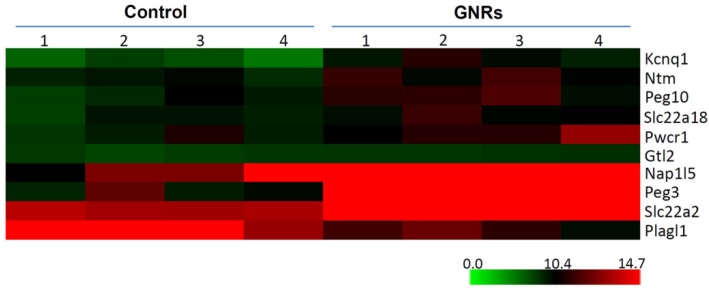
Differentially expressed imprinted genes in control and GNRs treated TM-4 Sertoli cells. The heat map indicates 10 differentially expressed imprinted genes in control and GNRs treated TM-4 Sertoli cells. Y axis indicates the names of the differentially expressed imprinted genes.

We finally tested relative expression levels of total 44 homologous imprinted genes (Table S1) according to the combining of mouse and human imprinted genes listed in the two databases. The names of tested imprinted genes in both mouse and human were listed in Table 1. From these genes, eleven (*Cdkn1c*, *Hymai*, *Igf2*, *Magel2*, *Mest*, *Ndn*, *Ppp1r9a*, *Sgce*, *Slc22a3*, *Snurf*, *Zim2*) of them were not found to be expressed in control or GNRs treated group (Table 1). Four imprinted genes (*Ano1*, *Nap1l5*, *Peg3*, *Slc22a2*) were expressed in control but not expressed in GNRs treated group (Table 1). In addition, *Plagl1* was found to be expressed in GNRs treated group but not in control (Table 1). The remaining 28 imprinted genes were expressed in both control and GNRs treated group. The Ct values of these 28 expressed imprinted genes ranged from 14.97 (*Gnas*) to 34.11 (*Magi2*) (Figure 2). Housekeeping gene *Gapdh* (mean Ct = 20.76 cycles) and U6 (mean Ct = 17.55 cycles) were used as references to compare expression of imprinted genes. The expression level of *Gnas* was the highest both in control and GNRs treated group. Of the expressed 28 imprinted genes, six (*Gtl2*, *Kcnq1*, *Ntm*, *Peg10*, *Pwcr1*, *Slc22a18*) were significantly differentially expressed (Table 2), and all of them were down-regulated (Figure 2). For the four imprinted genes which were expressed in control but not in GNRs treated group, we set the Ct values of the GNRs treated group as 40, and then studied the difference between the two groups. The result indicated that three (*Nap1l5*, *Peg3* and *Slc22a2*) of them showed statistical significance. However, *Ano1* did not show significance (*p* = 0.083). So we also regarded these three genes as differentially expressed, and they were down-regulated. Then we used the same method for *Plagl1*. We found the difference also showed statistical significance (Figure S1, Table 2). The expression level of *Plagl1* was significantly increased in GNRs treated group. Therefore, we identified 10 aberrantly expressed imprinted genes in GNRs treated group compared with control (Figure 3).

Since *Kcnq1* was the most differentially expressed imprinted gene of the 28 imprinted genes both expressed in control and GNRs treated TM-4 Sertoli cells, we studied the correlation between *Kcnq1* and other 5 (*Ntm*, *Peg10*, *Slc22a18*, *Pwcr1*, *Gtl2*) imprinted genes both expressed in control and GNRs treated TM-4 Sertoli cells. We found that the expression levels of *Ntm* (*r* = 0.714, *p* = 0.047), *Peg10* (*r* = 0.810, *p* = 0.015), *Slc22a18* (*r* = 0.905, *p* = 0.002) and *Pwcr1* (*r* = 0.738, *p* = 0.037) were positively correlated with *Kcnq1*.

### 3.2. Different Expression of Imprinted Genes between Expression Allele

**Figure 4:**
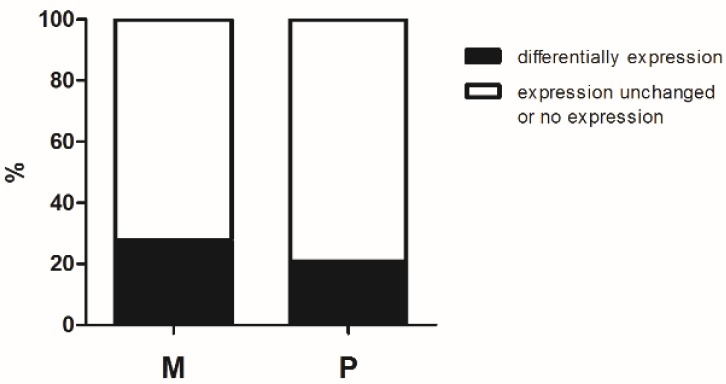
Ratio of differentially expressed imprinted genes between the maternal and paternal expressed imprinted genes. The results are presented as the percentage of differentially imprinted genes in maternal and paternal expressed imprinted genes respectively. Y axis indicates the expression ratio (represented as %). The maternal differentially expression ratio was 27.77% (5/18) and the paternal differentially expression ratio was 20.83% (5/24). *p* = 0.720 using Fisher’s Exact Test. P, paternal expressed genes; M, maternal expressed genes.

Of the tested 44 imprinted genes, eighteen were maternal expressed, twenty-four were paternal expressed and two were isoform dependent (Table S2). From the 10 aberrantly expressed imprinted genes, five were maternal expressed and five were paternal expressed (Table 2). Then we tested the ratio of differentially expressed imprinted genes between the maternal and paternal expressed imprinted genes. We found that there was no frequency difference between expression allele (Figure 4) (*p* = 0.720).

### 3.3. Enriched Biological Trends within the Imprinted Gene Set

We compared the location of the 10 imprinted genes, and found that four (*Kcnq1*, *Slc22a18*, *Pwcr1* and *Peg3*) of them were located on chromosome 7 in mouse and three (*KCNQ1*, *NTM*, *SLC22A18*) of them were located on chromosome 11 in human (Table 2), which suggested to us that the differentially expressed imprinted genes may have enriched biological trends. We used DAVID v6.7 (david.adcc.ncifcrf.gov) to analyze the gene list including 44 imprinted genes we tested. We found that there was a cluster including four imprinted genes (*Ano1*, *Sgce*, *Kcnk9* and *Slc22a2*) in 44 imprinted genes. Among the four imprinted genes, *Slc22a2* was significantly differentially expressed in GNRs treated group.

## 4. Discussion

GNR have showed promising applications in cancer diagnostics, treatment, drug delivery and the food industry, such as food packaging and beverages. They are often administered into circulation via direct injection. So, it is necessary to evaluate the adverse impact of GNRs. Our previous study revealed that GNRs cause reproductive toxicity in males by inducing the decrease of glycine synthesis, membrane permeability and mitochondrial membrane potential in TM-4 Sertoli cells [12]. Many studies demonstrated that chemicals could change the expression of imprinted genes in mouse [24] and human [25]. However, whether GNRs could affect the expression of imprinted genes remains unknown. Therefore, in this study, we tested the expression of 44 imprinted genes to explore whether GNRs could change the expression of imprinted genes in TM-4 Sertoli cells. In our earlier study, we found that GNRs treatment did not affect cell viability under the concentration of 10 nM while GNRs with higher dose caused decreased cell viability [12]. Neither exposure for 24 h nor 48 h reduced cell viability. Therefore, in the present study, we treated TM-4 cells with GNRs at 10 nM for 24 h to determine the expression of imprinted genes. 

First of all, we recruited 44 homologous imprinted genes between mouse and human. So the results for mice are analogous to the results from humans. Probably because imprinting is tissue-specific [19], eleven of 44 genes were neither expressed in control nor in treated group of TM-4 Sertoli cells. We found that 10 of the remaining 33 imprinted genes were changed by GNRs, nine of 10 were down regulated (*Kcnq1*, *Ntm*, *Peg10*, *Slc22a2*, *Pwcr1*, *Gtl2*, *Nap1l5*, *Peg3* and *Slc22a2*) while *Plagl1* was up regulated. Among these 10 imprinted genes, half were maternal expressed and half were paternal expressed. Maternal and paternal imprinted genes play different roles [26], paternal expressed (maternally imprinted) were accounted to promote growth while maternal pressed (paternally imprinted) genes were regarded as growth suppressors. So we tested whether GNRs have trend to influence different alleles. However, there was no significant difference between expression alleles. 

Many imprinted genes were found in clusters [27]. According to our results, four of the 10 differentially expressed imprinted genes were located on chromosome 7 in mouse and three were on chromosome 11 in human, this phenomenon is consistent with previous studies [28,29]. Deletions at and around the albino locus on chromosome 7 of the mouse induced by radiation could cause sterility in mice [30], which suggests us that mouse chromosome 7 might be strongly related to male infertility. In human, it has been ensured that chromosomal 11 region is associated with male factor infertility due to impaired spermatogenesis with and without cryptorchidism [31]. In the meantime, we further analyzed the enriched biological trends. We found that there was a cluster including four imprinted genes (*Ano1*, *Sgce*, *Kcnk9* and *Slc22a2*) in 44 imprinted genes and found that *Slc22a2* was significantly differentially expressed. *Ano1* was also differentially expressed, but the difference was not statistically significant.

Among these imprinted genes, microRNAs (miRNAs) are a class of small non-coding regulated RNAs. It has been reported that miRNA play important roles in spermatogenesis [32]. Nano-sized materials could also deregulate the expression of miRNAs [33]. So in the present study, we also tested the expression levels of three miRNAs belong to the imprinted genes in TM-4 cells after treatment with GNRs, although there were no significant differences.

Our results were consistent with early studies. *KCNQ1/KCNE1* is involved in K^+^ transport, they are associated in testis and their expression is regulated in development [34]. *KCNQ1* has also been reported in association with poor semen parameters or male infertility [35]. *PEG10* was found to be associated with placenta function and induce preeclampsia [36] and spontaneous miscarriages [37]. *SLC22A18* was reported to be associated with lifetime reproductive traits in pig [38]. Besides, the different 5-hydroxymethylcytosinepatterns of *SLC22A18* were found in globozoospermia sperm by using the method of sequencing of 5hmC-enriched genomic DNA [39]. *GTL2/MEG3* was found differentially expressed between infertile males and fertile controls, and the methylation differences showed significant effect [40]. *MEG3* and *PEG3* were also found to be changed in control and methoxychlor (MXC) treated group in sperm, and the methylation status were also changed [41]. *Peg3* was found to be associated with reduced litter sizes [42]. *KCNQ1* and *PEG3* were most significantly changed in our present study. These studies indicated that the 10 differentially expressed imprinted genes induced by GNRs were involved in reproduction, and different methylation pattern may be associated with the expression of imprinted genes. These results suggest that GNRs could potentially affect male reproduction via changing the expression of imprinted genes, and these imprinted genes might have joint roles in affecting reproduction.

## 5. Conclusions 

In conclusion, we revealed for the first time that GNRs exposure could cause aberrant expression of imprinted genes in TM-4 Sertoli cells. Future study is needed to investigate the mechanism of aberrant expression of imprinted genes in TM-4 Sertoli cells treated with GNRs and potential association between imprinted genes and glycine synthesis. Hence, these findings remind us that abnormal expression of imprinted genes might be an underlying mechanism for GNRs induced reproduction toxicity.

## Figures and Tables

**Table 1:** List of the imprinted genes examined in TM-4 Sertoli cells.

Gene		Expression		Gene		Expression		Gene		Expression	
Mouse	Human	0 nM	10 nM	Mouse	Human	0 nM	10 nM	Mouse	Human	0 nM	10 nM
*Ano1*	*ANO1*	Yes	No	*Kcnk9*	*KCNK9*	Yes	Yes	*Phlda2*	*PHLDA2*	Yes	Yes
*Cdkn1c*	*CDKN1C*	No	No	*Kcnq1*	*KCNQ1*	Yes	Yes	*Plagl1*	*PLAGL1*	No	Yes
*Ddc*	*DDC*	Yes	Yes	*Klf14*	*KLF14*	Yes	Yes	*Ppp1r9a*	*PPP1R9A*	No	No
*Dlk1*	*DLK1*	Yes	Yes	*Magel2*	*MAGEL2*	No	No	*Pwcr1*	*PWCR1*	Yes	Yes
*Gnas*	*GNAS*	Yes	Yes	*Magi2*	*MAGI2*	Yes	Yes	*Rian*	*MEG8*	Yes	Yes
*Gnas-as*	*GNAS-AS*	Yes	Yes	*Mest*	*MEST*	No	No	*Sgce*	*SGCE*	No	No
*Gpr1*	*GPR1*	Yes	Yes	*Mir296*	*MIR296*	Yes	Yes	*Slc22a18*	*SLC22A18*	Yes	Yes
*Grb10*	*GRB10*	Yes	Yes	*Mir298*	*MIR298*	Yes	Yes	*Slc22a2*	SLC22A2*	Yes	No
*Gtl2*	*MEG3*	Yes	Yes	*Mkrn3*	*MKRN3*	Yes	Yes	*Slc22a3*	SLC22A3*	No	No
*H19*	*H19*	Yes	Yes	*Nap1l5*	*NAP1L5*	Yes	No	*Snrpn*	*SNRPN*	Yes	Yes
*Hymai*	*HYMAI*	No	No	*Ndn*	*NDN*	No	No	*Snurf*	*SNURF*	No	No
*Igf2*	*IGF2*	No	No	*Nnat*	*NNAT*	Yes	Yes	*Tfpi2*	*TFPI2*	Yes	Yes
*Igf2as*	*IGF2AS*	Yes	Yes	*Ntm*	*NTM*	Yes	Yes	*Ube3a*	*UBE3A*	Yes	Yes
*Igf2r*	*IGF2R*	Yes	Yes	*Peg10*	*PEG10*	Yes	Yes	*Zim2*	*ZIM2*	No	No
*Inpp5f V2*	*INPP5F V2*	Yes	Yes	*Peg3*	*PEG3*	Yes	No				

Note: Names in bold indicates the genes were expressed both in GNRs treated group and control.

**Table 2:** Information of differentially expressed imprinted genes.

Gene	*p* Value	Location		Full Name	Gene Type	Expression Allele
		Mouse	Human			
*Kcnq1*	0.003	7 69.3 cM	11p15.5	potassium voltage-gated channel, subfamily Q, member 1	PC	M
*Ntm*	0.026	9 AS	11q25	neurotrimin	PC	M
*Peg10*	0.030	6 0.5 cM	7q21	paternally expressed 10	PC	P
*Slc22a18*	0.042	7 69.5 cM	11p15.5	solute carrier family 22 (organic cation transporter), member 18	PC	M
*Pwcr1*	0.046	7 29.0 cM AS	15q11.2	Prader-Willi syndrome chromosome region 1	NC	P
*Gtl2*	0.048	12 54.0 cM	14q32	gene trap locus 2	NC	M
*Nap1l5*	0.005	6 C1 AS	4q22.1 AS	nucleosome assembly protein 1-like 5	PC	P
*Peg3*	<0.001	7 6.5 cM AS	19q13.4 AS	paternally expressed 3	PC	P
*Slc22a2*	0.013	17 7.32 cM	6q26 AS	solute carrier family 22 (organic cation transporter), member 2	PC	M
*Plagl1*	0.019	10 15.0 cM	6q24-q25 AS	pleiomorphic adenoma gene-like 1	PC	P

Note: PC, protein coding; NC, non-coding; P, Paternal; M, Maternal.

## Supplementary Materials

The following are available online at www.mdpi.com/1660-4601/13/3/271/s1, Figure S1: Expression profiles of *Ano1*, *Na1pl5*, *Peg3*, *Slc22a2* and *Plagl1*, Table S1: List of primers used to test the expression of the 44 imprinted genes and the reference genes *Gapdh* and *U6*, Table S2: List of 44 homologous imprinted genes.

## Abbreviations

The following abbreviations are used in this manuscript:

GNRs: Gold Nanorods
ICR: Imprinting Control Regions
MXC: Methoxychlor
