# Supplementary material for: Effects of Gold Nanorods on Imprinted Genes Expression in TM-4 Sertoli Cells
Source: Int J Environ Res Public Health. 2016 Mar 1;13(3):271. doi: 10.3390/ijerph13030271 (PMC4808934; doi:10.3390/ijerph13030271)
Supplement: Supplementary file 1 [file ijerph-13-00271-s001.pdf]

Supplementary Materials: Effects of Gold Nanorods on Imprinted Genes Expression in TM-4 Sertoli Cells

Beilei Yuan, Hao Gu, Bo Xu, Qiuqin Tang, Wei Wu, Xiaoli Ji, Yankai Xia, Lingqing Hu, Daozhen Chen and Xinru Wang

Table S1. List of primers used to test the expression of the 44 imprinted genes and the reference genes Gapdh and U6.

Gene	Primer-F (5′-3′)	Primer-R (5′-3′)
Ano1	CTGATGCCGAGTGCAAGTATG	AGGGCCTCTTGTGATGGTACA
Cdkn1c	CCCATCTAGCTTGCAGTCTCTT	CAGACGGCTCAGGAACCATT
Ddc	TGGGGACCACAACATGCTG	TCAGGGCAGATGAATGCACTG
Dlk1	AGCTGCACCCCCAACC	CTGCTGGCGCAGTTGGTC
Gnas	TGCAAGGAGCAACAGCGAT	GCGGCCACAATGGTTTCAAT
Gpr1	GCTGGGAGTTGTTCACTGGG	GACGATGGCATTTCCTGGAAT
Grb10	AACCCAGCTTTTGCAGGAA	TCAGGGAGAAGATGTTGCTGT
Gtl2	GTTTCTGGACTGTGGGCTGT	CAACAGCAACAAAACTCAGAACATTCA
H19	GCACCTTGGACATCTGGAGT	TTCTTTCCAGCCCTAGCTCA
Hymai	TGCCTTTCAGTGTTGAACCA	TGCATCCCTAAACAACAGCTT
Igf2	AGCCGTGGCATCGTTGAG	GACTGCTTCCAGGTGTCATATTG
Igf2as	TCTTTGCCCTCTTTCGTCTC	CTCCAGGTGCTTCCGTCTAG
Igf2r	CTGCCGCTATGAAATTGAGTGG	CGCCGCTCAGAGAACAAGTT
Inpp5f V2	ACTGAACCTGAGCAGATTTCCA	CCACCCCACTCCAAAAGGTT
Kcnk9	GATGAAACGCCGGAAGTC	GTGTTCGGCTTTGGCAGT
Kcnq1	GCGTCTCCATCTACAGCACG	GAAGTGGTAAACGAAGCATTTCC
Klf14	TTTCCCTCACACTTGATTACCC	AGCGAGGGAGGGACTAAGAT
Magel2	GGGCTCCGCTAAATCATTG	CCCCTGCGGTCTATAGAAGA
Magi2	GGACTAGCAGGGTTCACGAA	GCTCCGACGTACGGAAACT
Mest	TGACCACATTAGCCACTATCCA	CCTGCTGGCTTCTTCCTATACA
Mir296	ACACTCCAGCTGGGAGGGCCCCCCCTCAA	CTCAACTGGTGTCGTGGAGTCGGCAATTCAGTTGAGACAGGATT
Mir298	ACACTCCAGCTGGGAGCAGAAGCAGGGAGGTT	CTCAACTGGTGTCGTGGAGTCGGCAATTCAGTTGAGTGGGAGAA

Table S1. *Cont.*

Gene	Primer-F (5′-3′)	Primer-R (5′-3′)
Mkrn3	GACCTTCCCGTCTGTGAGC	ATGTGGCAGGGCTGAATCT
Nap1l5	TTATCGAGAGCCTGCCTAATTCG	GCTTCTATCTTATCGCATCGCTT
Ndn	CTGATGATGTGTGTTGGGGTA	GGCTTTGCTGGTGACTTCTT
Nespas	ACTGAACCTGAGCAGATTTCCA	CCACCCCACTCCAAAAGGTT
Nnat	GTACATCTTCCGCGTGCTG	CTTCTCGCAATGGGCTGT
Ntm	CTCGCGTGGTCCTTCTGAG	TCTTTGGGTGGTTGTCTGTCT
Peg10	AAATTGCCTGACATGAAGAGGAGTCTA	AAGCCTAGTCACCACTTCAAAACACACTAAA
Peg3	ACATTTCTGGTGTTTGAGGAGTT	AGACCAGGTTCCGGTAATTCT
Phlda2	TCCATCCTCAAGGTGGACTG	ATCTCCTTGTGGTCGGTGGT
Plagl1	CATATTTGCATGTTAGAAGAATCAGC	TGAGTCAGTTAGGTCAGTGTAGAGAGA
Ppp1r9a	TGTACGATGGCCCTTCATATTCC	AGCGGTTGTTCTGTCCTGATT
Pwcr1	TGCTCTTTAGCAGGATGGTGT	GATGCAGGGATGCTCGTC
Rian	TCCAGTACCTGTTTGCCACA	GGGTAATGAGGTCCGGAAGC
Sgce	ATGGAGTTTCGCTCCTGTTG	GAGTCTGATGTGGCAAGTTCC
Slc22a18	CCTCAGCTTCACCTGCATCC	GTGTACCTGGCGGGGCTTAC
Slc22a2	GGGTCACTCTTCCTCATGCC	GCAGTAAGCCCTCTCCTTGG
Slc22a3	GAGCGCTTGTTCTGGTCTCA	TGCAGCTATCACACAAGCCA
Snrpn	GAGGAGTTGGGGGACCAT	CAGCTGCTACAGTGCCTCTTC
Snurf	TCTGATTCCAAGCAAAAACCA	GTTCTCCTAGAAACAATGCAAGC
Tfpi2	CTGGGGCTGTCGATTCTGC	TCTCCGCGTTATTTCCTGTTG
Ube3a	CGAATGGCCACAGCTTGTA	GCTTCATTCGGCTAGCTTCA
Zim2	TGCAAGGATTGTGGTAAGTCC	AGCTGCTGCTGCTTCATCTT
Gapdh	GCACCGTCAAGGCTGAGAAC	GGATCTCGCTCCTGGAAGATG
U6	CTCGCTTCGGCAGCACA	AACGCTTCACGAATTTGCGT
URP	TGGTGTCGTGGAGTCG	

Table S2. List of 44 homologous imprinted genes.

Gene	Location		Full Name	Gene Type	Expressed Allele
	Mouse	Human			
Ano1	7 AS	11q13.3	anoctamin 1, calcium activated chloride channel	PC	M
Cdkn1c	7 69.49 cM AS	11p15.5 AS	cyclin-dependent kinase inhibitor 1C (P57)	PC	M
Ddc	11 7.0 cM AS	7p12.2 AS	dopa decarboxylase	PC	P
Dlk1	12 54.0 cM	14q32	delta-like 1 homolog (Drosophila)	PC	P
Gnas	2 104.0 cM	20q13.3	GNAS (guanine nucleotide binding protein, alpha stimulating) complex locus	PC	ID
Gpr1	1 AS	2q33.3 AS	G protein-coupled receptor 1	PC	P
Grb10	11 8.0 cM AS	7p12-p11.2 AS	growth factor receptor bound protein 10	PC	ID
Gtl2	12 54.0 cM	14q32	gene trap locus 2	NC	M
H19	7 69.03 cM AS	11p15.5 AS	H19, imprinted maternally expressed transcript	NC	M
Hymai	10	6q24.2 AS	hydatidiform mole associated and imprinted transcript	NC	P
Igf2	7 69.09 cM AS	11p15.5 AS	insulin-like growth factor 2	PC	P
Igf2as	7 69.09 cM	11p15.5	insulin-like growth factor 2, antisense	NC	P
Igf2r	17 7.35 cM AS	6q26	insulin-like growth factor 2 receptor	PC	M
Inpp5f V2	7 F3	10q26.11	inositol polyphosphate-5-phosphatase F	PC	P
Kcnk9	15 D3 AS	8q24.3 AS	potassium channel, subfamily K, member 9	PC	M
Kcnq1	7 69.3 cM	11p15.5	potassium voltage-gated channel, subfamily Q, member 1	PC	M
Klf14	6 A3.3 AS	7q32.3 AS	Kruppel-like factor 14	PC	M
Magel2	7 28.0 cM	15q11-q12 AS	melanoma antigen, family L, 2	PC	P
Magi2	5	7q21 AS	membrane associated guanylate kinase, WW and PDZ domain containing 2	PC	P
Mest	6 7.5 cM	7q32	mesoderm specific transcript	PC	P
Mir296	2 AS	20q13.32 AS	microRNA 296	NC	P
Mir298	2 AS	20q13.32 AS	microRNA 298	NC	P
Mkrn3	7 29.0 cM AS	15q11-q13	makorin, ring finger protein, 3	PC	P
Nap1l5	6 C1 AS	4q22.1 AS	nucleosome assembly protein 1-like 5	PC	P
Ndn	7 28.0 cM	15q11.2-q12 AS	necdin	PC	P
Nespas	2 104.0 cM AS	20q13.32 AS	neuroendocrine secretory protein antisense	NC	P
Nnat	2 88.0 cM	20q11.2-q12	neuronatin	PC	P
Ntm	9 AS	11q25	neurotrimin	PC	M

Peg10	6 0.5 cM	7q21	paternally expressed 10	PC	P
Peg3	7 6.5 cM AS	19q13.4 AS	paternally expressed 3	PC	P
Phlda2	7 69.5 cM AS	11p15.5 AS	pleckstrin homology-like domain, family A, member 2	PC	M
Plagl1	10 15.0 cM	6q24-q25 AS	pleiomorphic adenoma gene-like 1	PC	P
Ppp1r9a	6 0.5 cM	7q21.3	protein phosphatase 1, regulatory (inhibitor) subunit 9A	PC	M
Pwcr1	7 29.0 cM AS	15q11.2	Prader-Willi syndrome chromosome region 1	NC	P
Rian	12 60.41 cM	14q32.31	RNA imprinted and accumulated in nucleus	NC	M
Sgce	6 1.0 cM AS	7q21-q22 AS	sarcoglycan, epsilon	PC	P
Slc22a18	7 69.5 cM	11p15.5	solute carrier family 22 (organic cation transporter), member 18	PC	M
Slc22a2	17 7.32 cM	6q26 AS	solute carrier family 22 (organic cation transporter), member 2	PC	M
Slc22a3	17 7.31 cM AS	6q26-q27	solute carrier family 22 (organic cation transporter), member 3	PC	M
Snrpn	7 29.0 cM AS	15q11.2	small nuclear ribonucleoprotein N	PC	P
Snurf	7 B5 AS	15q12	SNRPN upstream reading frame	PC	P
Tfpi2	6 1.0 cM AS	7q22 AS	tissue factor pathway inhibitor 2	PC	M
Ube3a	7 28.65 cM	15q11-q13 AS	ubiquitin protein ligase E3A	PC	M
Zim2	7 A1 AS	19q13.4 AS	zinc finger, imprinted 2	PC	M

Note: PC, protein coding; NC, non-coding; P, Paternal; M, Maternal; ID, isoform dependent.

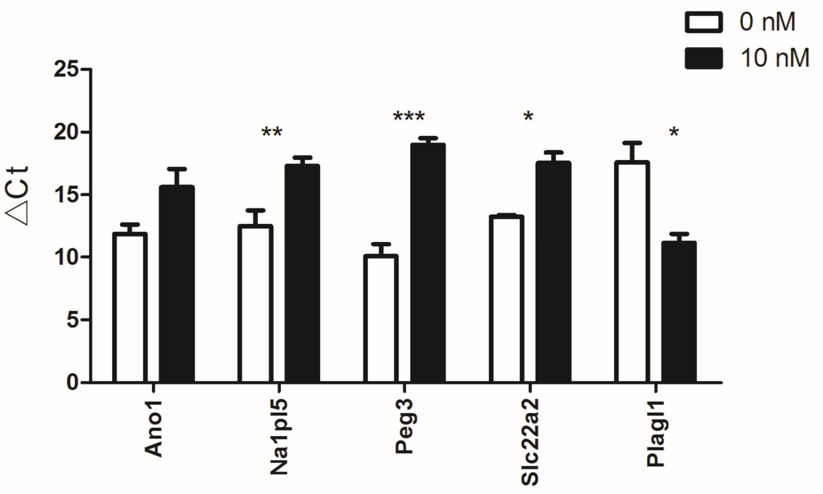

Figure S1. Expression profiles of *Ano1*, *Na1pl5*, *Peg3*, *Slc22a2* and *Plagl1*. Data are expressed as the mean ± SME. *$p < 0.05$; **$p < 0.01$; ***$p < 0.001$ versus control.

© 2016 by the authors; licensee MDPI, Basel, Switzerland. This article is an open access article distributed under the terms and conditions of the Creative Commons by Attribution (CC-BY) license (http://creativecommons.org/licenses/by/4.0/).
